# Acknowledgments

**DOI:** 10.4269/ajtmh.1121siii

**Published:** 2025-01-07

This supplement was produced with the support of the United States Agency for International Development (USAID) and the U.S. President’s Malaria Initiative (PMI) under the terms of the PMI Measure Malaria associate award 7200AA19LA00001. The views expressed herein are those of the author(s) and do not necessarily reflect the views of USAID or the United States Government.

We sincerely thank all the reviewers for dedicating their valuable time and expertise to reviewing the manuscripts and enhancing their quality.

The team would like to acknowledge the wonderful editorial team at AJTMH, especially Editor-in-Chief Phil Rosenthal, Managing Editor Alison Jaeb, Editorial Production Manager Annie Calacci, and Peer-Review Coordinator Lori Young.

Special thanks to Guest Editors Debra Prosnitz and Yazoumé Yé for overseeing the peer-review process.

